# Continuity of care in Klinefelter syndrome: age-adapted modules for standardized clinical data collection (I-KS)

**DOI:** 10.1530/EC-25-0196

**Published:** 2025-10-17

**Authors:** Corinna Grasemann, Claus H Gravholt, Lexi Breen, Lise Aksglaede, Angela Lucas-Herald, Malika Alimussina, Claudia Boettcher, Joline Wernsmann, Jens J Bauer, Jillian Bryce, Francesco Carlomagno, Sabine E Hannema, Andrea Isidori, Inas Mazen, Anna Nordenstroem, S Faisal Ahmed

**Affiliations:** ^1^ Department of Pediatrics, University Medical Center of The Johannes Gutenberg University Mainz, Mainz, Germany; ^2^ Department of Pediatrics, Katholisches Klinikum Bochum, Ruhr-University Bochum, Bochum, Germany; ^3^ Department of Endocrinology and Department of Molecular Medicine, Aarhus University Hospital, and Department of Clinical Medicine, Aarhus University, Aarhus, Denmark; ^4^ University of Lincoln, Brigg, United Kingdom; ^5^ Department of Growth and Reproduction, Copenhagen University Hospital Rigshospitalet, Copenhagen, Denmark; ^6^ Developmental Endocrinology Research Group, University of Glasgow, Glasgow, United Kingdom; ^7^ Inselspital University Hospital Bern Children’s Clinic, Julie-Von-Jenner Haus, Paediatric Endocrinology and Diabetology, University of Bern, Bern, Switzerland; ^8^ Department of Experimental Medicine, ‘Sapienza’ University of Rome, Rome, Italy; ^9^ Endo-ERN Centre ‘Azienda Ospedaliero-Universitaria Policlinico Umberto I’, Rome, Italy; ^10^ Department of Paediatrics, Amsterdam UMC Location Vrije Universiteit Amsterdam, Amsterdam, The Netherlands; ^11^ National Research Centre, Cairo, Egypt; ^12^ Pediatric Endocrinology, Karolinska University Hospital, Stockholm, Sweden

**Keywords:** Klinefelter syndrome, life span, age-adapted modules, transition, registry

## Abstract

**Abstract:**

Klinefelter syndrome (KS) is an underdiagnosed condition, affecting approximately 1 in 600 male births. Despite its relatively high prevalence, more than two-thirds of affected individuals remain undiagnosed, and clinical awareness is limited. KS presents with a highly variable phenotype, requiring lifelong, multidisciplinary care that spans pediatric and adult specialties. However, care is often fragmented, and there is no standardized approach to transitioning individuals from pediatric to adult healthcare services. Structured, longitudinal data collection is essential to better understand KS across the lifespan and to facilitate the transition process. To address this need, a group of clinical experts (pediatric and adult specialists) and patient representatives developed structured, age-adapted modules for longitudinal clinical data collection in KS. Through an iterative consensus process, a list of clinical, biochemical, diagnostic, and therapeutic parameters was developed. Experts then systematically evaluated and prioritized these parameters based on clinical relevance and feasibility of collection in routine practice. The final modules are designed to guide standardized assessments across four key age groups: infancy, childhood, adolescence, and adulthood. The structured templates aim to support healthcare professionals in providing comprehensive, age-appropriate care while enabling systematic data collection for research. These modules provide a framework for tracking key clinical parameters during the transition from pediatric to adult care, ensuring continuity and optimizing long-term health outcomes for individuals with KS. Implementation of these modules in clinical registries will facilitate pooled analyses, helping to address unresolved clinical questions and improve care across the lifespan.

**Plain language summary:**

Understanding and improving care for people with Klinefelter syndrome: Klinefelter syndrome (KS) affects approximately 1 in 600 males but often remains undiagnosed. To improve lifelong care, experts developed structured data collection tools for different age groups. This approach enhances clinical care, supports research, and facilitates smoother transitions from pediatric to adult healthcare.

## Introduction

Klinefelter syndrome (KS) (1) is a rare and often underdiagnosed chromosomal condition affecting approximately 1 in 600 male births. Despite its relatively high prevalence, more than two-thirds of individuals with KS remain undiagnosed, largely due to the highly variable presentation and limited clinical awareness (2). Klinefelter syndrome is characterized by the presence of an extra X chromosome (47,XXY), mostly in non-mosaic form (3), leading to a broad range of physical, endocrine, neurodevelopmental, and metabolic manifestations (4, 5). Given the lifelong impact of KS, individuals require multidisciplinary care that spans both pediatric and adult healthcare services (6). However, the management of KS is often fragmented, and there is no standardized framework to guide the transition from pediatric to adult care (7). As individuals age, their medical needs shift, making structured, longitudinal data collection essential for optimizing health outcomes.

During childhood and adolescence, two key aspects dominate KS care: neurocognitive development (8) and pubertal development (9), with particular attention to ensuring the timely diagnosis of testosterone deficiency (10, 11). Many children with KS experience developmental delays, particularly in speech and language acquisition, which may be associated with difficulties in school, social challenges, and an increased risk of attention-deficit/hyperactivity disorder (ADHD) and autism spectrum traits (8, 12, 13). These neurocognitive challenges often necessitate early intervention with speech therapy, educational support, and psychological counseling. If KS is identified early in life, a multidisciplinary approach in childhood may be implemented. As puberty approaches, impending testosterone deficiency becomes a critical concern, as individuals with KS may exhibit incomplete pubertal development, and in most adolescents, gonadal failure becomes evident with elevated gonadotropins during late puberty (14). Timely initiation of testosterone replacement therapy (TRT) is essential to promote typical pubertal progression, support muscle and bone development, and improve psychosocial well-being, as well as to avoid sequelae due to hypogonadism.

In adulthood, the clinical landscape of KS shifts. While most adult patients with KS should already be established on TRT, many remain undiagnosed until adulthood, with the diagnosis only being uncovered when seeking evaluation for infertility. Infertility is a defining feature of non-mosaic KS and represents a major reason for delayed diagnosis, as affected individuals typically present with azoospermia (15). Beyond reproductive health, adult patients with KS face an increasing burden of metabolic, cardiovascular, and mental health comorbidities (16, 17, 18, 19, 20, 21). Studies have shown an elevated risk of obesity, insulin resistance, type 2 diabetes, dyslipidemia, and hypertension in KS, contributing to a higher prevalence of cardiovascular disease (2, 22). In addition, Klinefelter syndrome is associated with an increased risk of anxiety, depression, and, in some cases, psychotic disorders (23), further underscoring the need for comprehensive medical and psychological care throughout life. Sexual dysfunction is common despite TRT (24) but underreported, and multiple other organ systems may be affected by KS.

Despite the well-documented multisystem involvement of KS, there remains a lack of structured guidance for clinical management, particularly regarding the transition from pediatric to adult care. Young adults with KS may be lost to follow-up during this critical period, especially if TRT has not been established at the time of transfer, resulting in delayed or inadequate treatment.

To improve patient outcomes and harmonize clinical care, systematic data collection in clinical registries is essential. Registries enable the collection of standardized, real-world data on rare conditions, facilitate long-term follow-up, help identify disease patterns, assess treatment outcomes, and highlight challenges faced in resource-limited settings. They also may guide clinical practice based on expert consensus and facilitate international collaboration across different healthcare systems, helping researchers and clinicians generate evidence that can further guide best practices.

A successful example of such an approach is the international registries platform for rare conditions affecting sex development and maturation (SDMregistries), which has a dedicated registry for differences and disorders of sex development (I-DSD), congenital adrenal hyperplasia (I-CAH), Turner syndrome (I-TS), and hypogonadotropic hypogonadism (I-HH) (25) and has proven valuable in studying rare endocrine conditions. By pooling data across multiple centers, this platform has provided insights into the natural history, management, and outcomes of individuals with several overlapping conditions (25, 26). Applying a similar model to KS could help bridge current knowledge gaps, improve clinical care, and support the development of evidence-based treatment guidelines. Thus, establishing a dataset that could then be used to develop a dedicated registry for KS (i.e., I-KS) would allow for systematic tracking of key clinical parameters, facilitate research on long-term health outcomes, and ultimately improve the quality of care and transition planning for individuals with KS. The objective of the current study was to use a recently described process (27) to develop a consensus on a minimum dataset that could be collected in a routine clinical setting in people with KS.

## Methods

### Expert group formation

A multidisciplinary team of experts was formed to develop a standardized clinical data collection framework for Klinefelter syndrome (KS). The group included pediatric endocrinologists, adult endocrinologists, urologists, and rehabilitation specialists from the United Kingdom, Denmark, Sweden, Italy, Germany, Switzerland, the Netherlands, and Egypt. Selection criteria for participation included significant clinical and/or research experience with KS, involvement in rare disease registries, and prior contributions to guideline development. A patient representative was included to ensure that patient perspectives were incorporated throughout the process.

### Age-group definition and item generation

Given the evolving clinical presentation of KS over the lifespan, the group defined four key age segments to ensure age-appropriate data collection: infancy (0–2 years), childhood (3–11 years), adolescence/puberty (12–18 years), and adulthood (≥18 years).

This categorization reflects major developmental and clinical changes in individuals with KS, including early neurodevelopment, pubertal onset, and transition to adult care.

During the initial meeting (July 2023), the group agreed on an overarching methodology adapted from the GloBE-Reg project (27). Members proposed parameters of clinical, biochemical, diagnostic, and therapeutic relevance for KS management. These parameters were stratified by age group to address the distinct needs of infants, children, adolescents, and adults with KS. Duplicate items were consolidated, and the resulting preliminary dataset was prepared for expert rating.

### Delphi-like rating process

To prioritize and finalize the dataset, a Delphi-like consensus method was employed. Experts participated in two main rating rounds, followed by discussion of the results (in April 2024 and June 2024), with an optional third discussion for unresolved items.

The voting categories included ‘scope’, ‘importance’, and ‘ease of collection’. With regard to scope, each expert indicated whether an item was relevant for ‘pediatric only’, ‘adult only’, or ‘both’ age groups. For importance, each item was rated as ‘high’, ‘medium’, or ‘low’ based on their perceived clinical relevance for KS management. Finally, ease of collection was assessed using the same three-tier scale indicating how feasible it would be to collect each parameter in routine practice.

The cutoffs were chosen as follows for ‘importance’, at least 70% of experts had to rate the item as ‘high’ for it to be considered in the minimal dataset, whereas for ‘ease of collection’, at least 50% of experts had to rate the item as ‘high’ to ensure routine data capture was realistic.

Experts could add written comments in a shared spreadsheet, explaining reservations, clarifying context-specific challenges (e.g., missing birth records in adults), or suggesting modifications to response options. The items that failed to meet the cutoff or that showed ‘divergent’ ratings (e.g., ‘high’ relevance but ‘low’ feasibility) were flagged for re-run. These items underwent further discussion and re-voting in subsequent rounds to achieve consensus.

### Registry architecture and database management

After reaching consensus, the parameters were categorized by age group and aligned with existing registry structures. The age-adapted modules will be integrated into the existing SDMregistries architecture to ensure a uniform data structure and storage. The architecture allows for the creation of a specific KS database that can be analyzed both independently and in the context of other DSD conditions. Data will be collected in pseudonymized form to ensure data privacy and protection, while also enabling exchange between the participating centers.

The quality of the collected data is ensured through standardized data collection protocols and regular training of the participating centers. An ethics committee oversees compliance with data protection regulations and the ethical appropriateness of data collection and usage. Patients and/or their legal representatives provide informed consent to participate in the registry.

**Figure 1:**
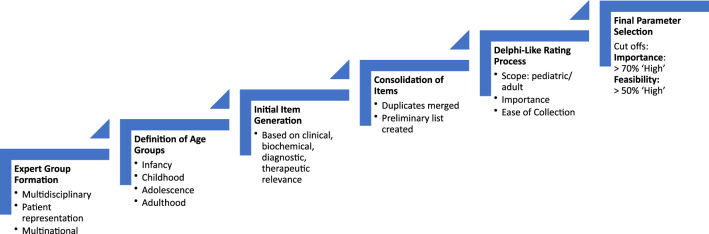
Flow chart of the process: formation of expert group, definition of age groups, item generation, item selection, iterative process, and cutoffs for final selection.

A flow chart of the process is provided in Fig. 1.

### Ethics statement

As this work involved the development of clinical data collection tools without the use of identifiable patient data or intervention, formal ethics approval was not required in accordance with institutional and national guidelines.

## Results

During the process, 302 parameters were initially suggested. After collation and merging of duplicates, a total of 161 parameters were sent to the group for rating of clinical importance and ease of collection.

A total of 55 parameters (core data: 6 items, infancy: 10 items, childhood: 13 items, adolescence/puberty: 19 items, adulthood: 7 items) were re-assessed due to divergent views of the panelists, mostly on ease of collection. All re-evaluated parameters were included in the final dataset, except for two: serum 17-hydroxyprogesterone (17OHP) and anogenital distance (AGD) in adults. While AGD was felt to be a worthy parameter, the panelists saw difficulties in obtaining it at routine clinical follow-up and agreed on adding it as a research-based parameter for adult individuals with KS.

As a result, a total of 159 parameters were agreed on, distributed over the following categories: demographics, diagnosis and past medical history, general health, bone health and body composition, gonadal function/reproductive function, puberty (Tanner stage) and maturation, laboratory tests, interventions and surgical events, therapies (medication/drugs/psychosocial/other), disclosure of condition/empowerment and transition.

The final list of parameters is presented as an overview in Table 1, showing the different parameters that are suggested for collection at initial assessment (i.e., core parameters) and in the different age groups (infancy, childhood, adolescence, and adulthood). A detailed list of parameters is provided in Supplementary Tables 2A and 2B (see section on Supplementary materials given at the end of the article), in which the individual parameters (Supplementary Table 2A) and the suggested mode of collection (units/response option) (Supplementary Table 2B) are provided.

**Table 1:** Summary of data elements collected across age groups. Overview of standardized clinical parameters selected via Delphi consensus for inclusion in the KS registry, organized by domain and stratified by age group. Elements reflect key diagnostic, therapeutic, metabolic, and psychosocial aspects relevant to longitudinal care.

Category	Parameter
Demographics	Date of birth
	Gender identity
	Gestational age, birth weight/length, head circumference
	Parental height
	Family history
Diagnosis & medical history	Karyotype, date/mode/reason for diagnosis/age at diagnosis
	Start/continuity of testosterone therapy (TRT)
	Associated conditions, past surgeries
	Participation in other registries/trials
General health	Education
	Employment status
	Living conditions
	Social support
	Auxology (height, weight, and BMI)
	Blood pressure
	Associated diagnoses
Bone health & body composition	DXA
	Percentage body fat/lean mass
Puberty & maturation	Tanner stage
	Skeletal age (X-ray)
Gonadal & reproductive function	Testis location/volume
	External genitalia
	Spontaneous erections/erectile dysfunction
	Fertility status, sperm analysis, mTESE, offspring
Laboratory tests	Testosterone, LH/FSH, inhibin, AMH, hemoglobin
	Thyroid function
	Adrenal function
	Glucose metabolism
	Lipid metabolism
	Bone metabolism
Therapy (medication)	Testosterone (details)
	Psychotropic medication
	Metabolic medication (e.g., statins, metformin, GLP-1 agonists)
	Anticoagulation
	Other
Psychosocial & supportive care	Quality of life
	Physical/occupational/speech therapy
	Educational/school support
	Social work, psychosocial support
	Knowledge of condition (child & parent)
	Age-appropriate disclosure of condition
	Support group contact
Transition planning	Transition readiness assessment

## Discussion

The development of this standardized framework for data collection in individuals with KS is an important step toward improving clinical care and research. By establishing a core set of parameters across different life stages, this dataset allows for a structured approach to evaluating the natural history of KS and current treatment practices, guiding clinical interventions, preparing for transitions of care in young individuals, and addressing unmet needs.

### Implications

The final set of 159 parameters covers a wide range of clinical, laboratory, therapeutic, and psychosocial aspects relevant to KS care. While extensive, this comprehensive dataset enables systematic monitoring and supports longitudinal studies. The selection process was adapted from the GloBE-Reg project (27) and ensured that parameters included in this dataset were both clinically relevant and feasible to collect.

The distribution of parameters across life stages aligns with the evolving medical and psychosocial needs of KS individuals. For example, pubertal assessments and gonadal function are emphasized during adolescence and puberty, reflecting the critical need to monitor the onset of testosterone deficiency (28). It is still being debated when testosterone treatment in adolescents should be commenced (4, 9), since serum levels of testosterone remain within the ‘normal range’ for a long time (28), despite elevation of gonadotropins and the development of clinical signs of hypogonadism (5, 29), e.g., excessive tiredness/sleep, anemia, depression (30), and osteopenia (31, 32). Using only testosterone levels to guide management on TRT in adolescents, therefore, results in delayed treatment of hypogonadism with detrimental effects on overall (33, 34) and mental health (30). Recent data show that appropriate testosterone supplementation reduces mortality substantially in comparison with untreated KS (35).

Similarly, psychosocial and transition-related parameters are highlighted in adolescence in this dataset, acknowledging the elevated stress levels during this period, as reported by Skakkebaek *et al.* (36). Of note, adolescents with rare conditions rarely indicate a need for psychosocial support themselves, as reported from a nationwide German project on transition care (37). However, previous research highlights higher rates of anxiety, depression, attention-deficit hyperactivity disorder (ADHD), schizophrenia (23), and social difficulties (38, 39) in individuals with KS. The inclusion of psychosocial assessments in the registry ensures that these aspects are monitored, allowing for early intervention and support.

There have been limited reports on gender identity within the KS population (40, 41), and it is currently not known whether gender dysphoria is increased within the KS community. As such, the use of estrogen as a potential hormone replacement therapy for individuals identifying as female has not been investigated. Knowledge in this field will expand as data are entered into registries, allowing for a better understanding of the frequency and impact of gender dysphoria in KS.

### Improving clinical care and bridging research gaps

By systematically collecting data in a uniform format, this framework aims to: i) enhance clinical care through standardized, age-appropriate assessments, ii) facilitate longitudinal studies on the natural history of KS and outcomes of interventions, and iii) streamline the transition of care from pediatric to adult healthcare services.

A strength of this registry is its ability to provide real-world data on individuals with KS across their lifespan, alongside current treatment modalities. This not only improves individualized patient management but also enables the identification of key clinical patterns and treatment responses and will allow visualization of clinical care in different European countries within the scope of the ERNs.

The registry will serve as a platform for descriptive and interventional research studies, to gain insights into the progression of KS, the effectiveness of treatment strategies, and quality-of-life outcomes. Regular reports and scientific publications generated from this dataset will contribute to evidence-based clinical guidelines.

By linking this dataset with the SDMregistries (42), it will allow for comparisons between KS and other conditions affecting sex development, providing a broader perspective on shared challenges and interventions.

### Transition readiness assessment

The transition from pediatric to adult care represents a critical period for individuals with a rare condition, including KS, and structured transition readiness assessments are essential in ensuring a smooth and effective transfer. Transition readiness tools, such as the transition readiness assessment questionnaire (TRAQ) (43, 44) or other validated instruments (37), provide a standardized method to evaluate patients’ preparedness in areas including medical self-management, self-advocacy, and independence in healthcare decisions. These can be used in parallel with the proposed framework for clinical data collection and may be especially useful in countries with limited resources. In addition, the harmonized dataset will facilitate a smooth transfer of care, with standardized key information being readily available for pediatric providers to share with adult providers.

Given that psychosocial stress is elevated during this phase (36), incorporating a structured transition assessment ensures that patients receive appropriate guidance and support tailored to their developmental stage. The inclusion of transition as a parameter in this registry, beyond recommendations for TRT (9), highlights the necessity of monitoring and addressing the challenges faced by adolescents and young adults with KS.

For adolescents and young adults with KS, transition and transfer of care occur at a pivotal time in life when TRT has been/will be initiated, and questions of sexual health and fertility are about to become increasingly relevant. Therefore, the empowerment to self-manage through the healthcare system, including these sensitive medical and psychosocial issues, is of utmost importance for young persons with KS.

### Patient empowerment and disclosure

Empowering individuals with KS through appropriate information disclosure is crucial in fostering self-efficacy and improving long-term health outcomes. Age-appropriate disclosure of the diagnosis and related health implications should be an integral part of KS management, allowing individuals to gradually understand their condition and actively participate in their care (45). Studies have shown that delayed or inadequate disclosure can lead to psychological distress (46), reduced adherence to treatment, and impaired self-management skills. Therefore, this registry includes parameters assessing how informed patients and parents/caregivers are over time, ensuring that their knowledge and self-management capabilities progress alongside their developmental needs. Structured interventions to support patient education, including shared decision-making strategies and peer support networks, may further enhance empowerment and engagement in care.

## Conclusion

With this dataset we provide a clear framework for clinically relevant parameters and investigations to facilitate comprehensive KS care. In addition, the registry will allow mapping of the current practice of hormone replacement therapy in KS. By incorporating key life–stage transitions and common comorbidities, this registry will serve as a valuable tool for clinicians, researchers, and patients of all ages. Moving forward, its successful implementation and continuous refinement will be crucial in optimizing health outcomes for individuals with KS.

## Supplementary materials



## Declaration of interest

The authors declare that there is no conflict of interest that could be perceived as prejudicing the impartiality of the work reported.

## Funding

This work has not received specific funding.
